# Subclinical steatohepatitis and advanced liver fibrosis in health examinees with nonalcoholic fatty liver disease (NAFLD) in 10 South Korean cities: A retrospective cross-sectional study

**DOI:** 10.1371/journal.pone.0260477

**Published:** 2021-11-24

**Authors:** Eun-Hee Nah, Seon Cho, Hyeran Park, Dongwon Noh, Eunjoo Kwon, Han-Ik Cho

**Affiliations:** 1 Health Promotion Research Institute, Korea Association of Health Promotion, Seoul, Korea; 2 MEDIcheck LAB, Korea Association of Health Promotion, Seoul, Korea; Osaka City University Graduate School of Medicine, JAPAN

## Abstract

**Background:**

Nonalcoholic steatohepatitis (NASH) has a risk of progressing to cirrhosis. The prevalence of NASH and its associated risk factors in community populations are relatively unknown. This study aimed to determine the prevalence of NASH and advanced liver fibrosis using magnetic resonance elastography (MRE), and determine those risk factors in health examinees with asymptomatic fatty liver.

**Methods:**

This study consecutively selected subjects who underwent health checkups at 13 health-promotion centers in 10 Korean cities between 2018 and 2020. Hepatic steatosis and stiffness were assessed using ultrasonography and MRE, respectively. Stages of liver stiffness were estimated using MRE with cutoff values for NASH and advanced liver fibrosis of 2.91 and 3.60 kPa, respectively.

**Results:**

The overall prevalence of NASH and advanced liver fibrosis in the subjects with fatty liver were 8.35% and 2.04%, respectively. Multivariate logistic regression analysis indicated that central obesity (OR = 5.12, 95% CI = 2.70–9.71), increased triglyceride (OR = 3.29, 95% CI = 1.72–6.29), abnormal liver function test (OR = 3.09, 95% CI = 1.66–5.76) (all *P*<0.001), and decreased high-density lipoprotein cholesterol (OR = 5.18, 95% CI = 1.78–15.05) (*P* = 0.003) were associated with NASH. The main risk factor for advanced liver fibrosis was diabetes (OR = 4.46, 95% CI = 1.14–17.48) (*P* = 0.032).

**Conclusion:**

NASH or advanced liver fibrosis is found in one-tenth of health examinees with asymptomatic fatty liver. This suggests that early detection of NASH should be considered to allow early interventions such as lifestyle changes to prevent the adverse effects of NASH and its progression in health examinees with asymptomatic fatty liver.

## Introduction

Nonalcoholic fatty liver disease (NAFLD) is the most common cause of liver disease worldwide [1,2]. Its prevalence increases alongside obesity and type 2 diabetes mellitus (T2DM) [3,4]. NAFLD patients have an increased risk of mortality from liver disease, and even more so from cardiovascular disease and malignancy [5].

NAFLD includes simple steatosis to nonalcoholic steatohepatitis (NASH), which can progress to cirrhosis in susceptible individuals. NASH was defined as presence of hepatic steatosis with inflammation and hepatocyte injury (ballooning) with or without fibrosis [6,7]. While simple steatosis is more benign, with an estimated risk of progressing to cirrhosis of lower than 4% in patients from the university hospital assessed with liver biopsy, NASH has an approximately 20% of risk the same progression [8,9]. It is important to identify NASH in patients to clinically manage the disease. Lifestyle changes in areas such as diet and exercise may improve histological findings in NASH patients [10].

Epidemiological studies assessing NASH prevalence and its associated risk factors are essential for designing effective screening strategies to diagnose NASH early. However, the epidemiology of NASH is relatively unknown in community subjects. The DIONYSOS study in northern Italy suggested that 43–55% of NAFLD patients with increased aminotransferase levels had histological evidence of NASH [11]. This study also derived from clinical assessments in patients with suspected liver disease. Two cohort studies suggested that 66% of diabetes or obesity patients older than 50 years had NASH with advanced fibrosis on the index liver biopsy [12,13]. NASH prevalence assessed by liver biopsies represents selected patients and does not reflect the prevalence within the community population.

Noninvasive techniques have been developed recently to replace liver biopsies. Magnetic resonance elastography (MRE) is a magnetic-resonance-imaging-based method for quantitatively determining liver stiffness (LS). Multiple studies have indicated that MRE-based LS measurements provide an accurate biomarker for detecting fibrosis [14–16]. MRE-based LS measurements could also discriminate between NASH and simple steatosis prior to fibrosis onset [17].

This study aimed to determine the prevalence of NASH and advanced liver fibrosis using MRE, and to determine NASH and advanced liver fibrosis risk factors in health examinees with asymptomatic fatty liver in 10 Korean cities.

## Materials and methods

### Study subjects and data collection

This was a cross-sectional retrospective study. Subjects who underwent health checkups including MRE and abdominal ultrasonography (US) were consecutively selected those from 13 health-promotion centers in 10 Korean cities across the country between January 2018 and June 2020. The 16 health-promotion centers belong to the Korea Association of Health Promotion, and comprise 3 centers in Seoul, 2 in Daegu, and 1 in each of Busan, Ulsan, Changwon, Incheon, Jeonju, Kwangju, Daejeon, Suwon, Chuncheon, Chungju, and Jeju. The National Health Insurance Service (NHIS) of Korea covers the entire population and provides biennial medical examinations. These 16 health-promotion centers perform approximately 10% of health checkups provided by the NHIS. Among the 16 health-promotion centers, 13 health-promotion centers that have MRE capabilities were selected for this study. Self-reported personal medical history and life style information for all participants were obtained at the time of health checkups. Exclusion criteria for this study included the presence of positive hepatitis B surface antigens or antibodies for hepatitis C, secondary causes of fatty liver (tamoxifen, antiobesity drugs and amiodarone), a history of hepatocellular malignancy, and excessive alcohol consumption defined as males and females consuming ≥14 and ≥7 standard units each week, respectively [18]. Analysis was performed on 4,303 of the 4,866 eligible subjects. The protocol of this study was reviewed and approved by the institutional review board of the Korea Association of Health Promotion (approval no. 130750-202009-HR-016). This study is a retrospective study of medical records and all data were fully anonymized before authors accessed them and IRB waived the requirement for informed consent.

### Fatty liver assessment using ultrasonography

The presence and degree of fatty liver were defined according to the results of abdominal US. Abdominal US was performed by standard criteria for diagnosing fatty liver based on parenchymal brightness, liver-to-kidney contrast, deep beam attenuation, and bright vessel walls. Severity is usually graded clinically using a four-point scale, as follows: normal (grade 0), mild (grade 1), moderate (grade 2), and severe (grade 3) [19].

### Liver stiffness measurements

All of the included subjects had undergone hepatic MRE examinations. MRE was performed using either MRE hardware (GE Healthcare, Waukesha, WI, USA) with a 1.5-T imaging system or a 1.5-T whole-body magnetic resonance unit (Gyroscan Intera, Philips Medical Systems, Best, the Netherlands) with a four-element torso coil. The two-dimensional MRE protocols used were similar to those described in previous literature [20,21]. The LS values of hepatic parenchyma were measured using MRE by drawing four regions of interest (ROIs) on the elastogram. ROIs were determined by the attending radiologists. All ROIs were drawn in areas indicated as having high confidence and good signal-to-noise ratio with stiffness outliers being excluded on the confidence map [22], and copied to corresponding positions on stiffness maps, providing LS values in kilopascals. After reconfirming the adequate placement of ROIs in the right liver lobe, LS values were calculated as the median value of multiple ROIs. The definition of NASH and advanced hepatic fibrosis were based on the MRE standard of 2.91–3.59 kPa and ≥3.60 kPa, respectively [17,23].

### Laboratory measurements

After an overnight fast, venous blood was drawn for health checkups that included the complete blood count (CBC), biochemical measurement, and Mac-2-binding protein glycosylated isomer (M2BPGi) levels. CBC and biochemical parameters were measured using the Sysmex XE-2100D analyzer (Sysmex, Kobe, Japan) and the Hitachi 7600 analyzer (Hitachi, Tokyo, Japan), respectively. Serum M2BPGi levels were measured using a chemiluminescence enzyme immunoassay (HISCL-5000, Sysmex, Kobe, Japan). APRI was calculated as AST (aspartate transaminase) (IU/L)/ULN (upper limit of normal)/platelet count (10^9^/L) × 100 [24]. The Fibrosis-4 Index (FIB-4) was calculated as the following formula: FIB-4 = age × AST (IU/L)/platelet count (10^9^/L) x √ALT (alanine aminotransferase) (IU/L) [25]. The fatty liver Index (FLI) is a noninvasive method of assessing hepatic steatosis using the body mass index (BMI), waist circumference (WC), triglyceride (TG), and gamma-glutamyl transferase (GGT), and is calculated using the following formula [26]: FLI = (*e*^0.953 × loge (TG) + 0.139×BMI + 0.718 × loge (GGT) + 0.053 × WC –15.745^)/(1 + *e*^0.953×loge (TG) + 0.139 × BMI + 0.718 × loge (GGT) + 0.053 × WC– 15.745^) × 100

The cutoffs for abnormal AST and ALT were >33 IU/L and >38 IU/L, respectively [27].

### Statistical analysis

All statistical analyses were performed using SAS version 9.4 (SAS Institute, Cary, NC, USA). Data were represented by mean ± standard deviation or frequency (percentage) values. The differences in the subject’s characteristics were analyzed according to the presence of fatty liver disease using Student’s *t*-test or the chi-square test. Univariate (crude) and multivariable (adjusted) logistic regression analyses were performed to identify the risk factors for fatty liver. Chi-square tests were used to compare the prevalence of steatohepatitis and advanced liver fibrosis according to age, BMI, fatty liver grades, and the presence of T2DM or metabolic syndrome. Differences between the four LS groups were analyzed using one-way ANOVAs and chi-square and Fisher’s exact tests. Univariate and multivariable logistic regression analyses were performed to identify the risk factors for steatohepatitis, and advanced liver fibrosis. *P* values of <0.05 were considered significant.

## Results

**Fig 1:**
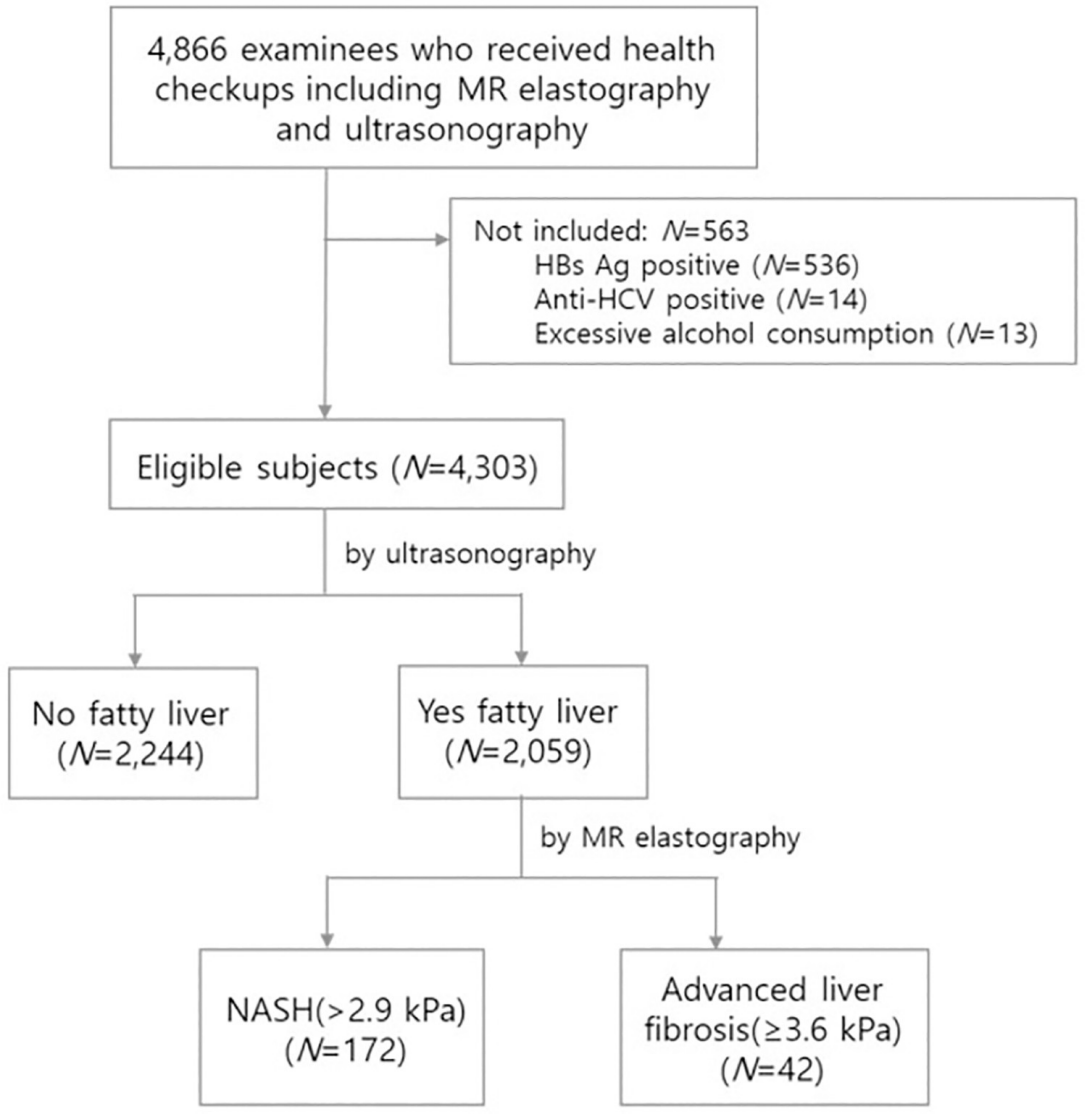
Flow chart of the study.

The 4,866 initially enrolled subjects who underwent health checkup including MRE and abdominal ultrasonography were consecutively selected from 13 health-promotion centers in Korea. After applying the exclusion criteria, 4,303 subjects were included in the study. Fatty liver was detected in 2,059 subjects by abdominal ultrasonography (Fig 1).

### Characteristics of the study subject

The age of all subjects was 47.4±10.4 years (range 20–84 years). The 4,303 study subjects included 2,059 (47.9%) with fatty liver detected by abdominal ultrasonography. Their mean BMI was 24.9 kg/m^2^. The prevalence of T2DM, hypertension, dyslipidemia, and metabolic syndrome among the study subjects were 11.4%, 21.3%, 38.5%, and 20.4%, respectively (Table 1). The subjects with fatty liver had higher BMI, higher presence rates of prediabetes, T2DM, hypertension, dyslipidemia, and metabolic syndrome, and higher levels of AST, ALT, GGT, M2BPGi, and platelet count. However, the subjects with fatty liver had lower AST/ALT ratio and FIB-4 than the subjects without fatty liver. The higher FIB-4 value in non-fatty liver group attributed that AST/ALT ratio was much higher in non-fatty liver group than in fatty liver group. In other word, ALT value in denominator of FIB-4 formula was much lower in non-fatty liver group.

**Table 1:** Characteristics of the study subjects.

Variable	All	Fatty liver (–)	Fatty liver (+)	P value
	(N = 4,303)	(N = 2,244)	(N = 2,059)	
Age, years	47.4 (± 10.4)	47.1 (± 11.1)	47.7 (± 9.7)	0.086
Sex, male	3,535 (82.2%)	1,680 (74.9%)	1,855 (90.1%)	<0.001
BMI, kg/m^2^	24.9 (± 3.18)	23.52 (± 2.66)	26.37 (± 3.03)	<0.001
WC, cm	85.6 (± 8.91)	81.52 (± 8.08)	89.95 (± 7.59)	<0.001
Prediabetes	1,660 (38.6%)	756 (33.7%)	904 (43.9%)	<0.001
T2DM	492 (11.4%)	127 (5.7%)	365 (17.7%)	<0.001
Hypertension	916 (21.3%)	347 (15.5%)	569 (27.6%)	<0.001
Dyslipidemia	1,658 (38.5%)	625 (27.9%)	1,033 (50.2%)	<0.001
Metabolic syndrome	865 (20.4%)	163 (7.4%)	702 (34.5%)	<0.001
Fatty liver grade				<0.001
Mild	1,334 (31.0%)	- (0.0%)	1,334 (64.8%)	
Moderate	672 (15.6%)	- (0.0%)	672 (32.6%)	
Severe	53 (1.2%)	- (0.0%)	53 (2.6%)	
AST, IU/L	30.4 (± 17.8)	27.0 (± 13.5)	34.1 (± 20.9)	<0.001
ALT, IU/L	32.3 (± 26.7)	24.0 (± 17.3)	41.3 (± 31.7)	<0.001
GGT, IU/L	56.8 (± 82.4)	44.6 (± 56.1)	70.1 (±102.2)	<0.001
AST/ALT ratio, %	1.11 (± 0.43)	1.26 (± 0.41)	0.95 (± 0.39)	<0.001
Platelet, 10^3^ μ/L	248.69 (±53.47)	246.22 (±54.14)	251.36 (±52.63)	0.002
FBS, mmol/L	5.5 (± 1.2)	5.3 (± 0.9)	5.8 (± 1.4)	<0.001
HbA1c, mmol/mol	39.4 (± 5.4)	38.0 (± 4.1)	40.7 (± 6.1)	<0.001
TC, mmol/L	5.2 (± 1.0)	5.1 (± 0.9)	5.3 (± 1.1)	<0.001
TG, mmol/L	1.67 (± 1.31)	1.28 (± 0.87)	2.09 (± 1.55)	<0.001
HDL-C, mmol/L	1.4 (± 0.3)	1.5 (± 0.3)	1.2 (± 0.3)	<0.001
LDL-C, mmol/L	3.1 (± 0.9)	3.1 (± 0.8)	3.1 (± 1.0)	0.005
MRE, kPa	2.31 (± 0.53)	2.26 (± 0.51)	2.37 (± 0.55)	<0.001
M2BPGi COI	0.6 (± 0.26)	0.57 (± 0.26)	0.63 (± 0.25)	0.049
Fatty liver index	40.7 (± 27.5)	27.2 (± 22.3)	55.3 (± 25.0)	<0.001
APRI	0.33 (± 0.25)	0.30 (± 0.19)	0.37 (± 0.29)	<0.001
FIB-4	1.13 (± 0.65)	1.16 (± 0.67)	1.10 (± 0.63)	0.008

Data are mean±standard deviation or N (%) values.

P value from t-test or chi-square test.

**Abbreviations** AST, aspartate transaminase; ALT, alanine aminotransferase; GGT, gamma–glutamyl transpeptidase; TC, total cholesterol; TG, triglycerides; HDL-C, high-density lipoprotein cholesterol; LDL-C, low-density lipoprotein cholesterol; MRE, magnetic resonance elastography; APRI, aspartate transaminase -to-platelet ratio index; FIB-4, Fibrosis-4 index; BMI, body mass index; WC, waist circumference; T2DM, type 2 diabetes mellitus, COI; cutoff index.

### Risk factors for fatty liver

In the univariate model, male sex, age, obesity, central obesity, impaired fasting glucose, hypertension, and dyslipidemia were risk factors for fatty liver (all *P*<0.05). In multivariable analysis, male sex [OR = 2.379, 95% CI = 1.946–2.908], age <50 years (OR = 1.860, 95% CI = 1.105–3.129), age 50–69 years (OR = 1.886, 95% CI = 1.124–3.165), central obesity (OR = 4.035, 95% CI = 3.457–4.711), hyperglycemia (OR = 1.699, 95% CI = 1.461–1.977), increased TG (OR = 2.414, 95% CI = 2.081–2.800), and decreased high-density lipoprotein cholesterol (HDL-C) (OR = 2.066, 95% CI = 1.694–2.521), as well as hypertension (OR = 1.351, 95% CI = 1.128–1.618) (all *P*<0.05) were associated with fatty liver (Table 2).

**Table 2:** Factors associated with fatty liver.

Variable	Univariate			Multivariable		
	OR	95% CI	P value	OR	95% CI	P value
Age						
<50	1.585	1.006–2.433	0.047	1.860	1.105–3.129	0.019
50–69	1.708	1.095–2.663	0.018	1.886	1.124–3.165	0.016
≥70	1			1		
Sex, male	3.053	2.567-,3.63	<0.001	2.379	1.946–2.908	<0.001
Obesity, BMI ≥25 kg/m^2^	5.208	4.573–5.932	<0.001	-	-	-
Central obesity*	5.47	4.738–6.314	<0.001	4.035	3.457–4.711	<0.001
Hyperglycemia*	2.396	2.104–2.728	<0.001	1.699	1.461–1.977	<0.001
Hypertension*	2.088	1.797–2.425	<0.001	1.351	1.128–1.618	0.001
TG, ≥1.69 mmol/L*	3.91	3.423–4.468	<0.001	2.414	2.081–2.800	<0.001
Decreased HDL-C*	2.698	2.272–3.205	<0.001	2.066	1.694–2.521	<0.001

* NCEP-ATPIII criteria.

Central obesity: Males, WC ≥90 cm; females, WC ≥85 cm.

Hyperglycemia: ≥5.6 mmol/L.

Hypertension: Systolic blood pressure≥130 or diastolic blood pressure≥85 or taking blood pressure medicine.

Decreased HDL-C: Males, ≤1.0 mmol/L; females, ≤1.3 mmol/L.

### Correlation between clinical prediction formulae for liver fibrosis and fatty liver grade

Estimation of correlations between fatty liver grade and clinical prediction formulae for liver fibrosis were also performed using FLI, APRI, and FIB-4. FLI and APRI were positive correlations with fatty liver grade but FIB-4 was negatively correlated with fatty liver grade (Table 3).

**Table 3:** Correlation between clinical prediction formulae and fatty live grade.

	Fatty liver grade	Fatty liver index	APRI	FIB-4
Fatty liver grade	1.000			
Fatty liver index	0.322**			
APRI	0.193**	0.300**		
FIB-4	-0.117**	-0.067*	0.575**	1.000

Correlation values (Rho) from Spearman’s rank correlation test.

* P<0.05,

** P <0.001.

### Presumed NASH without liver fibrosis and Presumed NASH with advanced liver fibrosis prevalence in fatty liver

Table 4 lists the prevalence of NASH (2.91–3.59 kPa) and advanced liver fibrosis (≥3.60 kPa) according to the characteristics of the subjects with fatty liver. The overall prevalence rates of NASH and advanced liver fibrosis in these subjects with fatty liver was 8.35% and 2.04%, respectively. NASH prevalence increased gradually with age, up to 12.5% among those aged ≥70 years (*P* = 0.022). NASH prevalence also increased with obesity grade, hyperglycemia, and the presence of metabolic syndrome (all *P*<0.001). However, fatty liver grades were not related to the prevalence of NASH. The prevalence of advanced liver fibrosis showed similar trends to those of NASH (Table 4).

**Table 4:** Prevalence of nonalcoholic steatohepatitis (NASH) and advanced liver fibrosis according to characteristics of the study subjects with fatty liver (N = 2,059).

Factor	Presumed NASH without liver fibrosis* (N = 172)		P value	Presumed NASH with advanced liver fibrosis* (N = 42)		P value
Overall	N = 172/2,059	(8.35%)		N = 42/2,059	(2.04%)	
Age group, years			0.022			0.480
<30	N = 3/33	(9.1%)		-	-	
30–39	N = 23/411	(5.6%)		N = 6/411	(1.5%)	
40–49	N = 52/738	(7.0%)		N = 12/738	(1.6%)	
50–59	N = 63/623	(10.1%)		N = 18/623	(2.9%)	
60–69	N = 27/222	(12.2%)		N = 5/222	(2.3%)	
≥70	N = 4/32	(12.5%)		N = 1/32	(3.1%)	
BMI, kg/m^2^			<0.001			<0.001
<18.5	-	-		-	-	
18.5–22.9	N = 7/230	(3.0%)		N = 7/230	(3.0%)	
23.0–24.9	N = 21/458	(4.6%)		N = 7/458	(1.5%)	
25.0–29.9	N = 109/1,131	(9.6%)		N = 14/1,131	(1.2%)	
≥30	N = 35/239	(14.7%)		N = 14/239	(5.9%)	
Fatty liver grade			0.079			0.658
Mild	N = 98/1,334	(7.3%)		N = 27/1,334	(2.0%)	
Moderate	N = 69/672	(10.3%)		N = 13/672	(1.9%)	
Severe	N = 5/53	(9.4%)		N = 2/53	(3.8%)	
T2DM			<0.001			<0.001
No	N = 39/790	(4.9%)		N = 4/790	(0.5%)	
Pre-T2DM	N = 86/904	(9.5%)		N = 13/904	(1.4%)	
Yes	N = 47/365	(12.9%)		N = 25/365	(6.8%)	
Metabolic syndrome			<0.001			0.032
No	N = 80/1,334	(6.0%)		N = 21/1,334	(1.6%)	
Yes	N = 92/702	(13.1%)		N = 21/702	(3.0%)	

Data are N (%) values.

P value from chi-square test.

*MRE liver stiffness values for “presumed NASH without liver fibrosis” and “presumed NASH with liver fibrosis” were 2.91–3.59 kPa and ≥3.60 kPa, respectively.

Metabolic syndrome: Conforming with three or more NCEP-ATPIII criteria.

### Clinical and laboratory characteristics of study subjects with fatty liver according to LS stage using MRE

Those with NASH or advanced liver fibrosis were predominantly older, male, and had higher BMI, AST, ALT, GGT, and fasting blood sugar levels, and higher presence of T2DM, hypertension, and metabolic syndrome compared with subjects with normal LS (*P*<0.001). In addition, liver fibrosis score such as APRI, FIB-4, and FLI as estimations of hepatic steatosis were also higher in those with NASH or advanced liver fibrosis than in those with normal LS (*P*<0.001) (Table 5).

**Table 5:** Clinical and laboratory characteristics of study subjects according to liver stiffness category using MRE in fatty liver.

Variable	Normal (<1.94 kPa)		Mild stiffness (1.94–2.90 kPa)		NASH (2.91–3.59 kPa)		Advanced liver fibrosis (≥3.60 kPa)		P value
Overall	N = 357	(17.3%)	N = 1,488	(72.3%)	N = 172	(8.4%)	N = 42	(2.0%)	
Age, years	47.4	(± 10 ^a^)	47.4	(± 9.6 ^a^)	50	(± 9.5^a^)	50.7	(± 9.0 ^a^)	0.002
Sex, male	N = 302	(84.6%)	N = 1,357	(91.2%)	N = 159	(92.4%)	N = 37	(88.1%)	0.002
BMI, kg/m²	25.8	(± 2.8 ^a^)	26.3	(± 3.0^a^^,^^b^)	27.8	(± 3.3 ^c^)	27.3	(± 4.5 ^b^^,^^c^	<0.001
Overweight, BMI ≥23 to <25 kg/m^2^	N = 94	(26.3%)	N = 336	(22.6%)	N = 21	(12.2%)	N = 7	(16.7%)	<0.001
Obesity, BMI ≥25 kg/m^2^	N = 206	(57.7%)	N = 992	(66.7%)	N = 144	(83.7%)	N = 28	(66.7%)	
Central obesity	N = 158	(44.3%)	N = 730	(49.1%)	N = 115	(66.9%)	N = 25	(59.5%)	<0.001
T2DM	N = 40	(11.2%)	N = 253	(17.0%)	N = 47	(27.3%)	N = 25	(59.5%)	<0.001
Hypertension	N = 91	(25.5%)	N = 397	(26.7%)	N = 62	(36.0%)	N = 19	(45.2%)	0.003
Dyslipidemia	N = 181	(50.7%)	N = 729	(49.0%)	N = 99	(57.6%)	N = 24	(57.1%)	0.142
Metabolic syndrome	N = 111	(31.8%)	N = 478	(32.5%)	N = 92	(53.5%)	N = 21	(50.0%)	<0.001
Fatty liver grade									0.314
Mild	N = 236	(66.1%)	N = 973	(65.4%)	N = 98	(57.0%)	N = 27	(64.3%)	
Moderate	N = 115	(32.2%)	N = 475	(31.9%)	N = 69	(40.1%)	N = 13	(31.0%)	
Severe	N = 6	(1.7%)	N = 40	(2.7%)	N = 5	(2.9%)	N = 2	(4.8%)	
Platelet count, ×10^9^/L	260.40	(± 50.20 ^b^)	250.50	(± 51.70 ^b^)	247.70	(± 57.90 ^b^)	220.60	(± 66.20 ^a^)	<0.001
AST, IU/L	31.3	(± 14.7 ^a^)	32.4	(± 16.3^a^)	42.9	(± 31.7^b^)	81.9	(± 58.5 ^c^)	<0.001
ALT, IU/L	36.9	(± 25.8 ^a^)	39.8	(± 27.0 ^a^)	53.7	(± 52.8 ^b^)	82.8	(± 62.6 ^c^)	<0.001
GGT, IU/L	57.5	(± 53.3 ^a^)	62.9	(± 58.2 ^a^)	89.2	(± 88.1 ^a^)	350.8	(± 508.6 ^b^)	<0.001
AST/ALT ratio, %	0.97	(± 0.31 ^a^)	0.94	(± 0.39 ^a^)	0.94	(±0.39 ^a^)	1.16	(± 0.66 ^b^)	0.002
FBS, mmol/L	5.7	(± 1.3 ^a^)	5.7	(± 1.3 ^a^)	6.1	(± 1.4 ^a^)	6.9	(± 2.1 ^b^)	<0.001
HbA1c, mmol/mol	39.4	(± 4.1^a^)	40.7	(± 6.1 ^a^)	42.1	(± 6.8 ^a^^b^)	43.4	(± 8.1 ^b^)	<0.001
TC, mmol/L	5.5	(± 1.1 ^a^)	5.3	(± 1.1 ^a^)	5.1	(± 1.0 ^a^)	5.3	(± 1.2 ^a^)	0.003
TG, mmol/L	2.01	(± 1.33 ^a^)	2.09	(± 1.61 ^a^)	2.26	(± 1.53 ^a^)	2.07	(± 1.29 ^a^)	0.395
HDL-C, mmol/L	1.3	(± 0.3 ^a^^,b^)	1.2	(± 0.3 ^a^)	1.2	(± 0.3 ^a^)	1.4	(± 0.5 ^b^)	0.002
LDL-C, mmol/L	3.3	(± 1.0 ^b^)	3.1	(± 1.0^a^^,b^	2.9	(± 1.0 ^a^)	3.0	(± 1.1 ^a^^,^^b^)	<0.001
M2BPGi COI	0.6	(± 0.21 ^a^)	0.63	(± 0.3 ^a^)	0.65	(± 0.3 ^a^)	0.67	(± 0.2 ^a^)	0.936
Fatty liver index	50.7	(± 24.6 ^a^)	54.5	(± 24.9 ^a^)	67.3	(± 22.1 ^b^)	73.2	(± 22.5 ^b^)	<0.001
APRI	0.33	(± 0.18^a^)	0.34	(± 0.18^a^)	0.47	(± 0.42 ^b^)	1.14	(± 1.11 ^c^)	<0.001
FIB-4	1.02	(± 0.45 ^a^)	1.06	(± 0.52 ^a^)	1.3	(± 0.78 ^b^)	2.48	(± 1.87 ^c^)	<0.001

Data are mean±standard-deviation or N (%) values.

^a,b,c,d^: Different letters indicate a significant difference between groups based on Scheffe’s multiple-comparisons test.

P value from one-way ANOVA or chi-square test.

### Factors associated with NASH and advanced liver fibrosis

Multivariable logistic regression analysis using significant variables from the univariate analysis (central obesity, T2DM, increased TG, decreased HDL-C, and abnormal liver function test [LFT]) indicated that the risk factors for NASH were central obesity (OR = 5.12, 95% CI = 2.70–9.71) increased TG (OR = 3.29, 95% CI = 1.72–6.29), raised liver transaminases (OR = 3.09, 95% CI = 1.66–5.76) (all *P*<0.001), and decreased HDL-C (OR = 5.18, 95% CI = 1.78–15.05) (*P* = 0.003). The risk factors for advanced liver fibrosis were age (OR = 0.42, 95% CI = 0.21–0.85) (*P* = 0.016) and T2DM (OR = 4.46, 95% CI = 1.14–17.48) (*P* = 0.032) (Table 6).

**Table 6:** Factors associated with NASH and advanced liver fibrosis in fatty liver from multivariable logistic regression analysis.

Variables	NASH*						Advanced liver fibrosis*					
	Univariate			Multivariable			Univariate			Multivariable		
	OR	95% CI	P value	OR	95% CI	P value	OR	95% CI	P value	OR	95% CI	P value
Age, per 10 years	1.04	0.81–1.34	0.771	1.11	0.78–1.60	0.560	0.47	0.27–0.81	0.006	0.42	0.21–0.85	0.016
Sex, male	1.16	0.48–2.81	0.745	0.67	0.19–2.42	0.545	1.73	0.50–5.99	0.390	0.58	0.11–3.00	0.519
Central obesity	7.52	4.26–13.27	<0.001	5.12	2.70–9.71	<0.001	4.58	1.73–12.09	0.002	2.39	0.73–7.84	0.149
Hypertension	1.61	0.94–2.75	0.084	0.87	0.44–1.75	0.700	1.53	0.62–3.78	0.362	1.82	0.51–6.59	0.359
Diabetes	3.97	1.85–8.50	<0.001	1.94	0.77–4.88	0.162	6.30	2.25–17.62	<0.001	4.46	1.14–17.48	0.032
TG, ≥1.69 mmol/L	4.35	2.55–7.41	<0.001	3.29	1.72–6.29	<0.001	2.27	0.88–5.88	0.091	2.27	0.67–7.70	0.190
Decreased HDL-C	7.73	2,97–20.14	<0.001	5.18	1.78–15.05	0.003	1.09	0.36–3.31	0.878	0.61	0.13–2.83	0.523
Raised liver transaminases^†^	3.78	2.22–6.43	<0.001	3.09	1.66–5.76	<0.001	4.01	1.27–12.69	0.018	2.13	0.55–8.29	0.276

^†^ Raised liver transaminases: AST > 33 IU/L and/or ALT > 38 IU/L.

*MRE liver stiffness values for “NASH” and “Advanced liver fibrosis” were 2.91–3.59 kPa and ≥3.60 kPa, respectively.

## Discussion

This study found that NASH and advanced liver fibrosis were present in 8.35% and 2.04%, respectively, of health examinees with fatty liver from health checkups. NASH is associated with central obesity, abnormal LFT and dyslipidemia conditions such as increased TG and decreased HDL-C, while advanced liver fibrosis is associated with T2DM.

Our study recruited asymptomatic individuals from the community population who underwent health checkups, not hospital patients. MRE is a non-invasive method that allowed accurate assessments of NASH and advanced liver fibrosis in many subjects within the community, where liver biopsies are less feasible.

The prevalence of NAFLD and NASH vary between countries, study populations, and their diagnostic modalities. The global prevalence of NAFLD is estimated to range from 17–46% in adults [28]. This is parallel to the prevalence of metabolic syndrome and its components [29], and has been rapidly increasing over the last decade [30]. NAFLD has become an issue more recently in Asian countries than in Europe or North America [31]. Industrialization in many Asian countries has increased sedentary behavior and over nutrition, which lead to obesity and metabolic disorders. A systemic review on NAFLD prevalence in Korea found that the prevalence was 30.3% (41.1% and 20.3% in males and females, respectively) [32]. The diagnostic modalities for NAFLD used in these studies were mostly based on ultrasonography. Many studies have also investigated NAFLD prevalence in Korea, and found that it ranged from 15 to 52% [33,34]. Our study indicated that the overall prevalence of NAFLD in Korea was 47.9% when using ultrasonography to diagnose fatty liver. The high proportion of males in our study and the higher prevalence of NAFLD in males could explain its higher prevalence compared with other Korean population studies.

Ultrasonography can identify fatty liver with moderate sensitivity, but it cannot distinguish between NASH and liver fibrosis. Liver biopsy is the current gold standard for NASH and liver fibrosis diagnosis and staging, but it is invasive and cannot be used in population-based studies. A Korean study on potential liver transplant donors found that the NAFLD prevalence was 51% in liver biopsies [35]. A study at the Brooke Army Medical Center in the US indicated that 46% of patients with fatty liver detected using ultrasonography had fatty liver in liver biopsy, among whom 30% and 7% had NASH and advanced liver fibrosis, respectively [36]. Since these patients were recruited from hospitals, those were not population studies. Our current study assessed NASH and advanced liver fibrosis using MRE. In our study, 8.35% and 2.04% of people with fatty liver in the community population had NASH and advanced liver fibrosis, respectively.

Several animal studies have indicated that increases in LS to where liver cell injury preceded the development of hepatic fibrosis [37]. Chen et al. [17] proposed a threshold of stiffness when using MRE to discriminate between NASH and simple steatosis. This discrimination is important to the clinical management of NAFLD. Diseases not related to NASH such as simple steatosis do not progress or progress very slowly, whereas NASH has a potentially progressive course toward liver disease [38]. Cryptogenic cirrhosis is currently considered to be burnt-out NASH [39]. Patients with early-stage NASH who suffer from inflammation but not fibrosis can adopt lifestyle interventions even when they have some degree of LS. In clinical practice, lifestyle changes including exercise and diet can be adopted as part of NASH therapy. A prospective study of a dietary intervention in patients with biopsy-confirmed NASH indicated that a 10% decrease in body weight has histological benefits [40]. In addition, weight reduction ameliorated both hepatic fat deposition and liver stiffness associated with NAFLD in obese children [41].

While NASH prevalence increases with older age, age was not an independent risk factor for NASH in this study. The relationship between age and liver fibrosis in NAFLD is controversial. Vernon et al. [28] indicated that the relationships of NAFLD prevalence and fibrosis with age in NAFLD may be related to the duration of the disease rather than to age itself.

NAFLD has a close association with insulin resistance not only in the liver, but also in muscle and adipose tissues, and with metabolic syndrome [42]. In our study, metabolic syndrome and its components–including central obesity, increased TG, and decreased HDL-C–were associated with NASH. Advanced liver fibrosis was closely associated with T2DM. These findings could be explained by previous studies. Hepatic TG accumulation occurs in parallel with abnormal hepatic energy metabolism [43], impaired insulin-mediated hepatic glucose suppression, and the production of very low-density lipoprotein [44], leading to hyperglycemia and hypertriglyceridemia. Prediabetes was also closely associated with fatty liver, NASH, and advanced fibrosis in our study, which was consistent with previous studies [4,28]. Meanwhile, lean NASH was also found in our study. The proportion of patients with NASH was higher when compared between the groups with and without diabetes among the lean patients with NAFLD among our cohort in our another study [45]. Lean patients with NAFLD who have a lower number of metabolic risks showed a non-negligible prevalence of diabetes similar to that in non-lean patients with NAFLD. The presence of diabetes was the most specific predictive factor for hepatic fibrosis in lean patients with NAFLD.

Our study has some limitations. First, fatty liver was only assessed using ultrasonography. Although ultrasonography can detect fat deposition in the liver, it is a subjective diagnosis of fatty liver and cannot assess the disease severity. Second, selection bias may have been present due to different reasons for seeking health checkups, such as MRE and abdominal ultrasonography. MRE and abdominal ultrasonography were additionally performed on subjects in our study who voluntarily underwent MRE and sonography examinations during health checkups. It is possible that those who are more concerned about their hepatic condition underwent the MRE and abdominal ultrasonography examinations. This suggests that the study population may not be representative of the broader Korean population. In addition, there were more males than females in this study. This may have caused selection bias, which also suggests that the study population may not be representative of the broader Korean population. Third, though occult hepatitis B infection and HBeAg seroconversion are associated with disease progression to cirrhosis and hepatocellular carcinoma, authors could only exclude HBs Ag-positive subjects in exclusion of our study population as there were limited data about other markers of HBV such as HBV DNA, HBe Ag, and anti-HBe. Fourth, the cross-sectional design means that further research is needed to determine causal relationships. Nevertheless, the inclusion of a large, nationwide sample should have allowed some valid conclusions to be drawn from this study.

### Conclusions

NASH or advanced liver fibrosis is found in one-tenth of health examinees with asymptomatic fatty liver. NASH in particular comprises a large proportion of 8.35%, suggesting that its early detection should be considered to allow early interventions such as lifestyle changes to prevent the adverse effects of NASH and its progression in health examinees with asymptomatic fatty liver.

## Supporting information

S1 Data (XLSX)
